# Effects of definitive chemoradiation on circulating immunologic angiogenic cytokines in head and neck cancer patients

**DOI:** 10.1186/s40425-016-0138-9

**Published:** 2016-06-21

**Authors:** Vishwajith Sridharan, Danielle N. Margalit, Stephanie A. Lynch, Mariano Severgnini, F. Stephen Hodi, Robert I. Haddad, Roy B. Tishler, Jonathan D. Schoenfeld

**Affiliations:** Department of Radiation Oncology, Brigham and Women’s Hospital/Dana-Farber Cancer Center, 450 Brookline Ave, DA L2-57, Boston, MA 02114 USA; Harvard-MIT Division of Health Sciences and Technology, Harvard Medical School, Boston, MA USA; Center for Immuno-oncology, Dana-Farber Cancer Institute, Boston, MA USA; Department of Medical Oncology, Brigham and Women’s Hospital/Dana-Farber Cancer Center, Boston, MA USA

**Keywords:** Angiogenesis, Cytokines, Radiation, Head and neck cancer, VEGF, PLGF, Angiopoietins

## Abstract

**Background:**

Preclinical studies suggest a synergistic effect between radiation, immunotherapy and anti-angiogenic therapy, although the mechanisms are unclear. Angiogenic cytokines are known to affect the immune system, and their levels may be associated with response to immunotherapy. Here, we assess changes in circulating VEGF, as well as angiogenic cytokines angiopoietin-1 and -2 (Ang1, Ang2), and placental growth factor (PLGF) that occur during definitive chemo-radiotherapy in HNSCC patients.

**Methods:**

We prospectively collected blood samples from patients receiving definitive radiation with or without chemotherapy. Serum Ang1, Ang2, VEGF, and PLGF were measured via cytokine assays.

**Results:**

The majority of patients had advanced stage, node positive HPV-associated oropharyngeal cancer, and received radiation to a median dose of 70 Gy with concurrent cisplatin. Over the course of treatment, serum VEGF and Ang1 levels decreased in 20/24 (84 %, *p* < 0.0001) and 21/24 (88 %, *p* < 0.0001) patients, respectively, and Ang2 and PLGF levels increased in 20/24 (83 %, *p* < 0.0001) patients.

**Conclusions:**

We find significant changes in angiogenic cytokines in the majority of HNSCC patients over the course of chemoradiation. Decreases in VEGF caused by radiation may represent one mechanism of potential synergy with immunotherapy. Increases in Ang2 and PLGF are interesting given their link to tumor associated angiogenesis and poor prognosis. Additional studies are needed to explore synergies between anti-angiogenic treatments, immunotherapy, and chemoradiation in HNSCC.

**Electronic supplementary material:**

The online version of this article (doi:10.1186/s40425-016-0138-9) contains supplementary material, which is available to authorized users.

## Background

Head and neck squamous cell carcinoma (HNSCC) affects greater than 500,000 people across the world annually, and ranks 6^th^ in global cancer incidence [1]. Tobacco and alcohol use are the predominant risk factors for tumor development worldwide, but the oncogenic human papilloma virus (HPV) 16, has also been implicated in disease pathogenesis, particularly in developed countries [2]. Multimodality treatment, including a combination of surgery, chemotherapy, and radiation therapy (RT) is individualized for patients with head and neck cancers, with many patients receiving radiation either with or without chemotherapy as definitive treatment [3]. Despite recent treatment advances with more refined surgical techniques such as transoral robotic surgery (TORS), and more precise intensity-modulated radiation (IMRT), there remain many subgroups of patients (heavy-tobacco smokers, or those with heavy nodal burden) who develop local recurrences or distant metastases, with five-year overall survival less than 50 % [4].

Tumor-associated angiogenesis is an established target for therapy, with anti-angiogenic agents being actively investigated for the treatment of multiple malignancy types [5]. Tumor angiogenesis is a complex process whereby endothelial cells transition from a quiescent to an active state in part because of cytokine signals received from malignant cells. This process is intricately related to tumor associated inflammation, immune evasion, and metastasis [6, 7]. Several key regulators of tumor angiogenesis have been identified, including vascular endothelial growth factor (VEGF), placental growth factor (PLGF), and angiopoietins-1 and 2 (Ang1, Ang2) [8, 9]. VEGF also serves important immunomodulatory roles, including inhibition of dendritic cell maturation, expansion of regulatory T-cells (T-regs) and myeloid derived suppressor cells (MDSCs), downregulation of effector T-cells and may also specifically impact anti-tumor immunity [10–13]. Similar to VEGF, Ang2 can also affect tumor immunity by augmenting the expression of immunosuppressive cytokines (i.e, IL-10) in monocytes and macrophages, and promote the expansion of T-regs [14]. Immunologic effects of Ang1 and PLGF are less well defined in comparison to VEGF and Ang2.

Over 90 % of HNSCC express VEGF and other angiogenic cytokines, which serve as attractive treatment targets [15, 16]. The anti VEGF antibody, bevacizumab, has demonstrated a survival benefit across several malignancies and has also demonstrated early promising results in head and neck cancer, producing responses in both the primary and metastatic setting [5, 17–19]. Ang1 and Ang2 were also overexpressed within tumor deposits in a study of 40 patients undergoing surgical resection of recurrent oral squamous cell carcinoma (OSCC), and high expression of these angiogenic cytokines was correlated with nodal involvement and more advanced disease [20]. Similarly, a separate study examining 100 OSCC tumor specimens found higher PLGF expression was associated with lymph node spread and more advanced clinical stage [21].

Anti-angiogenic therapies can aid in sensitizing tumors to radiation by normalizing abnormal tumor vasculature associated with hypoxic radioresistant regions [22, 23]. Preclinical evidence in various cancers also suggests that RT may impact tumor vasculature and perhaps anti-tumor immunity in part by altering levels of angiogenic cytokines in various cancers, but clinical data is limited [24, 25]. Here, we prospectively assess changes in circulating Ang-1, Ang-2, PLGF, and VEGF as a result of definitive chemo-radiotherapy in HNSCC patients, and identify tumor- and treatment-specific trends that could aid in developing future combination regimens.

## Methods

### Study design and patient population

We prospectively enrolled consecutive head and neck cancer patients receiving definitive intent head and neck radiation with or without chemotherapy on a Dana-Farber/Harvard Cancer Center Institutional Review Board-approved protocol to collect longitudinal peripheral blood samples. In order to be eligible, all patients needed to have gross disease above the clavicles that was being targeted by radiation treatment and were without evidence of metastatic disease. All patients provided informed consent.

We collected baseline information from all patients including demographics, histology, disease site, as well as tumor and nodal stage. For squamous cell carcinomas originating in the oropharynx or nasopharynx, we determined association with the human papilloma virus (HPV), using both in situ hybridization for high-risk HPV types (16 and 18), and immunohistochemistry for the p16 protein.

All patients were prescribed a 7-week course of curative-intent radiation with or without chemotherapy. All patients received radiation that was graphically planned, and with the exception of one early stage larynx cancer patient, all patients were treated to the primary site and bilateral neck with intensity modulated radiation therapy (IMRT) to maximize normal tissue sparing. Radiation was delivered daily Monday-Friday; all patients were evaluated at least once weekly by the treating radiation oncologist or more often if clinically warranted. Blood samples were obtained in phlebotomy just before the beginning and end of therapy. Blood samples were processed within 2 h of draw. Sample processing took 30 min, and samples were stored on ice from time of collecting till processing. PBMCs and plasma were stored at –80C.

Following the completion of treatment, all patients were followed regularly in multidisciplinary head and neck clinic. Restaging positron emission tomography/computed tomography (PET-CT) and CT of the head and neck was performed approximately 3 months after treatment or earlier if clinically indicated. Neck dissection was performed if there was suspicion for residual disease at the three month time period. We abstracted treatment details and information about clinical course and potential recurrence from the medical record.

### Cytokine assays

We isolated serum from blood samples using centrifugation (3000g, 10 min, 4C) and then stored these samples at –80C. Samples that were collected were frozen immediately at –80C upon centrifugation. Samples were then thawed and aliquoted for subsequent assays, that were then run the same day when possible. No assay was conducted with 3 or more thaw cycles, and data for a particular cytokine was only compared among a uniform number of freeze thaw cycles. We prospectively hypothesized that chemoradiation would impact circulating levels of angiogenic cytokines, and therefore serum from a homogenous group of consecutive patients with squamous cell carcinoma were assayed for Ang2, VEGF, and PLGF levels using the Bio-Plex Human Cancer Biomarker Panel (Biorad Laboratories Inc, Hercules, CA). Ang1 levels were assayed using the Magnetic Luminex Screening Assay (R&D Systems Inc, Minneapolis, MN). All samples were tested as experimental repeats, and measured against a standard curve of purified protein, according to the manufacturer’s protocol [26]. Luminex systems were quality assured via “Bio-Plex System Validation and Calibration Kits” (Bio-Rad) to test the instrument prior to every assay. Moreover, the instrument is cleaned before and after every per manufacturer protocol. Fluorescence intensity was measured via the Bio-Plex MAGPIX Multiplex Reader (Biorad Laboratories Inc, Hercules, CA).

### Flow cytometry

We isolated peripheral blood mononuclear cells (PBMCs) via centrifugation (1500 g, 20 min), and stored the PBMCs in freezing media (10 % FBS RPMI + 10 % DMSO) at –80C on the same day of the blood draw. Flow cytometry was performed to quantify CD4+, CD8+ (CD3-PC7+, CD4-FITC+/CD8-APC+) and myeloid-derived suppressor cell populations (MDSCs, CD14-APC+, HLA-DR PC7-) via established protocols. All antibodies were obtained from eBioscience (San Diego, CA) except for CD8 APC (Miltenyi Biotec Inc, San Diego, CA). FlowJo (Ashland, OR) was used for analysis.

### Statistical methods

Correlations between cytokine levels and tumor and treatment parameters including sex, age, site of primary disease, HPV status, nodal involvement, smoking status were evaluated using the *χ*^2^ test or pairwise Student’s t-test. We compared changes in cytokine levels at the beginning and end of therapy using non-parametric Wilcoxon signed rank tests. Two-sided *p*-values < .05 were considered statistically significant and Bonferroni corrections for multiple testing were performed where indictated. All statistical analyses were computed using GraphPad Prism (GraphPad Software Inc, La Jolla, CA), or JMP Pro12 (SAS Institute Inc, Cary, NC).

## Results

### Patient characteristics and treatment parameters

We initially evaluated 24 consecutive patients with squamous cell carcinoma whose blood samples were assessed for angiogenic cytokines. Baseline patient characteristics are displayed in Table 1. The median age of patients was 58 years (IQR 52.5–66). The majority of patients were male (92 %) with locally-advanced human papilloma virus (HPV)-associated oropharyngeal cancer. The majority of patients presented with nodal involvement, with either one (*n* = 7) or two (*n* = 12) positive nodes. Eleven (46 %) had smoking history of greater than 10 pack-years. Median radiation dose delivered was 70 Gy; only one patient received less than this dose (64 Gy). The majority of patients received concurrent chemotherapy with radiation (*n* =21, 88 %). Eleven patients (52 % of patients that received chemotherapy) received concurrent bolus cisplatin at 100 mg/m2 every three weeks, eight (38 %) received weekly cisplatin chemotherapy, two (10 %) received weekly carboplatin-taxol. Median follow-up time for all patients was 10 months (range 5–17 months); there have been no pathologically proven local or distant failures to date.

**Table 1:** Patient characteristics

	Parameter	Number	Percent
Median Age, IQR		58, 52.5–66	
Sex			
	Female	2	8
	Male	22	92
Site			
	Base of tongue	15	63
	Tonsil	4	17
	Supraglottic larynx	1	4
	Nasopharynx	2	8
	Oral cavity	1	4
	Unknown primary	1	4
HPV Status			
	Positive	20	83
	Negative	2	8
	Not applicable/Unknown	2	8
Smoking			
	< 10 pack-years	13	54
	> 10 pack-years	11	46
Concurrent chemotherapy			
	Bolus cisplatin	11	46
	Weekly cisplatin	8	33
	Carboplatin-Taxol	2	8
	No chemotherapy	3	13
T-stage			
	Tx	1	4
	T1	8	33
	T2	7	29
	T3	5	21
	T4	3	13
N-stage			
	N0	5	21
	N1	4	17
	N2a	3	13
	N2b	10	42
	N2c	2	8
Stage			
	I	2	8
	II	2	8
	III	3	13
	IV	17	71

### Circulating angiogenic cytokines

Circulating angiogenic cytokines were measured at the beginning of treatment and then again the final week. Comparative analysis with a control donor patient pool at the same dilution as our cancer patients indicated that all angiogenic cytokines were present at a higher concentration in head and neck cancer patients. Median levels of VEGF, Ang1, Ang2 and PlGF at baseline were 0.46 ng/mL, 14 ng/mL, 0.33 ng/mL, and 0.16 ng/mL in HNSCC patients, respectively, as compared to undetectable, 0.42 ng/mL (*p* < 0.0001 compared to cancer patients), undetectable/below range, and undetectable (~0.001 ng/mL).

**Fig. 1:**
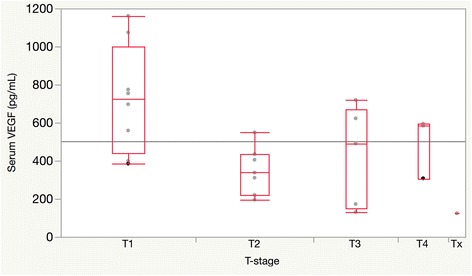
Increase serum VEGF levels at baseline are inversely correlated with tumor T-stage; *p*-values obtained via pairwise t-tests. T1 compared with T2 (*p* = 0.005), T1 compared with T3 (*p* = 0.03), T1 compared with T4 (*p* = 0.15), T2 compared with T3, T4 (*p* = 0.37)

There were no statistically significant associations between baseline levels of VEGF, the angiopoietins, or PLGF with sex, age at cancer diagnosis, disease subsite, HPV-disease status, presence of nodal disease, smoking status, or baseline red blood cell, white blood cell, platelet or monocyte serum levels (Additional file 1: Table S1). Earlier T-stage was associated with increased levels of baseline circulating VEGF (*p* = 0.0085, T1 compared with higher T-stage, Fig. 1).

**Fig. 2:**
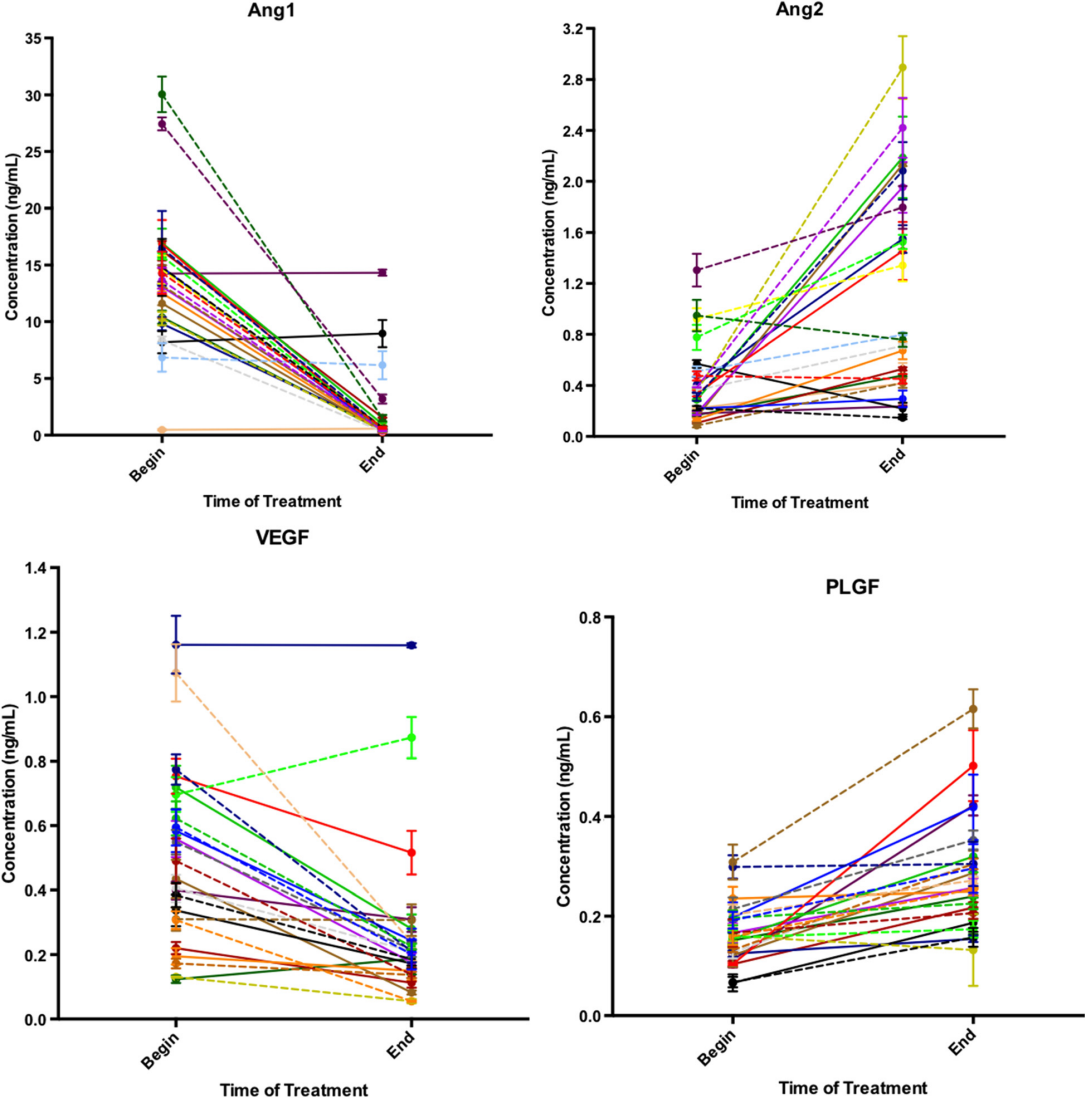
Trends in serum angiogenic cytokine concentrations comparing the beginning and end of treatment, as evaluated by non-parametric Wilcoxon signed rank tests. Each line represents an individual patient. Serum levels of Ang1 (*p* < 0.0001) and VEGF decreased (*p* < 0.0001) over the course of treament. In contrast, there were significant increases inserum levels of Ang2 (*p* < 0.0001) and PLGF (*p* < 0.0001)

Changes in angiogenic cytokine levels over the course of treatment are displayed in Fig. 2. Serum levels of Ang1 decreased in 21/24 (88 %) patients from a median 14 ng/mL (IQR 10–16) to a median 0.6 ng/mL (IQR 0.4–1.2, *p* < 0.0001). Circulating levels of VEGF also decreased in 20/24 (83 %) from a median 0.46 ng/mL (IQR 0.31–0.66) to 0.21 ng/mL (0.14–0.26, *p* < 0.0001). In contrast, serum levels of Ang2 and PLGF significantly increased. Median Ang2 levels were 0.33 ng/mL (IQR 0.18–0.49) prior to treatment, as compared with 0.78 after (IQR 0.44–1.9, *p* < 0.0001), with 20/24 (83 %) patients showing increased levels. PLGF similarly increased in 20/24 (83 %) patients from a median of 0.16 ng/mL (IQR 0.12–0.20) at the beginning of treatment to a median of 0.26 after treatment (IQR 0.21–0.31, *p* < 0.0001). All of these changes observed over the course of treatment remained statistically significant after adjusting for multiple testing.

**Fig. 3:**
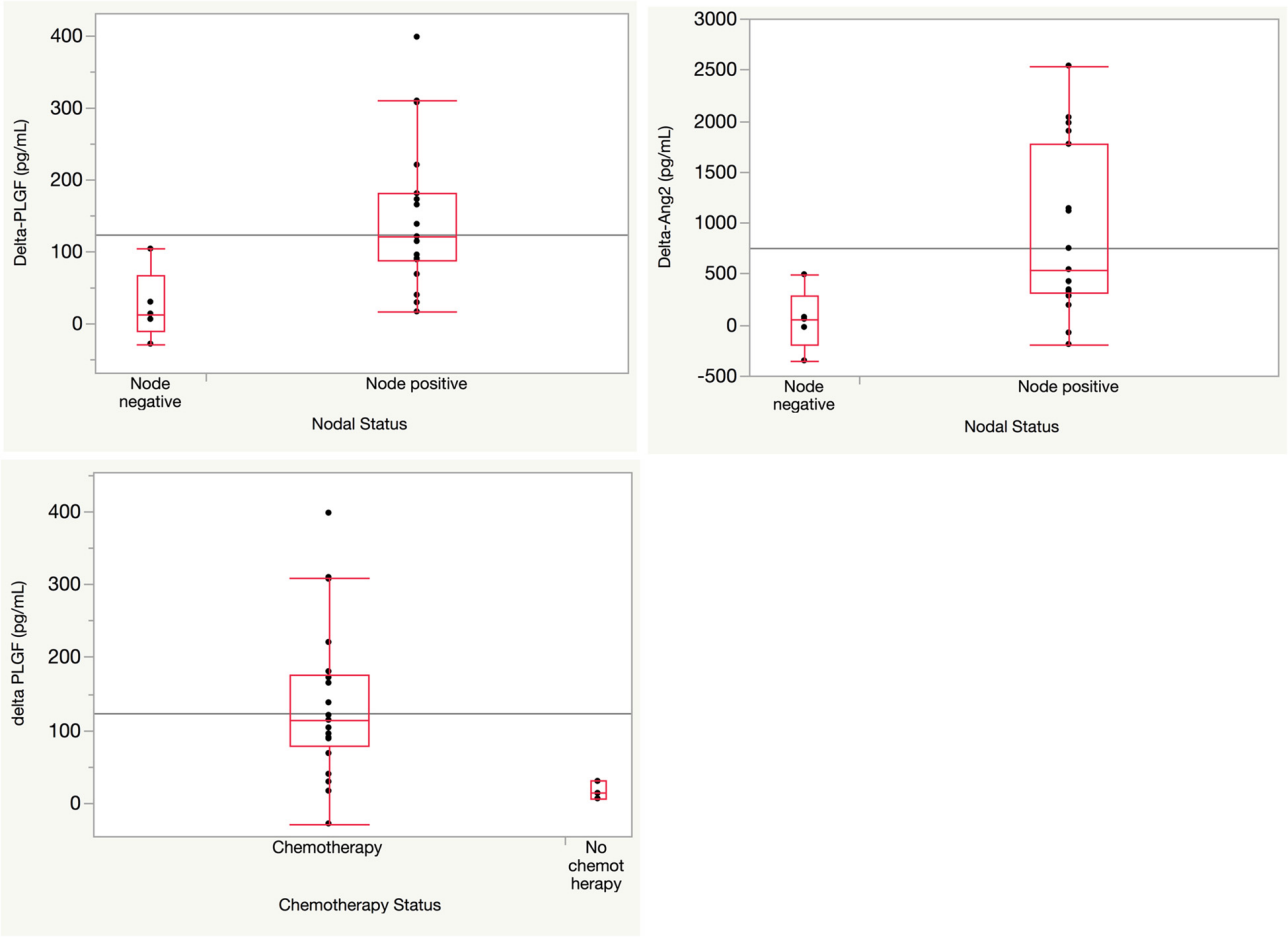
Correlations between tumor, treatment parameters and circulating angiogenic cytokines, as evaluated by non-parametric Wilcoxon signed rank tests. Changes in Ang2 (Delta-Ang2) and in PlGF (Delta-PlGF) values correlate with disease present in lymph nodes (*p* = 0.02, and 0.008, respectively, top panel). Delta-PLGF values also correlate with addition of concurrent chemotherapy (*p* = 0.02, bottom panel)

Overall stage and disease site were not associated with differential effects on any of the cytokines measured (data not shown). In contrast, nodal disease was associated with degree of change in Ang2 and PLGF levels over the course of treatment (Fig. 3). Node-negative patients demonstrate significantly less increase in Ang2 levels (median 0.057 ng/mL, IQR -0.19 to 0.28 ng/mL) compared to node-positive patients (median 0.54 ng/mL, IQR 0.31–1.8 ng/mL, *p* = 0.02). Similarly, increases in PLGF levels were lower in node-negative patients (median 0.013 ng/mL, IQR -0.011–0.066) compared to node-positive patients (median 0.12 ng/mL, IQR 0.088–0.18, *p* = 0.008). These changes were no longer statistically significant after adjusting for multiple testing. Similar correlations were not observed with or changes in Ang1 (*p* = 0.75) or changes in VEGF (*p* = 0.80).

In addition, we found a potential effect of concurrent chemotherapy on changes in the levels of specific circulating angiogenic cytokines (Fig. 3). Patients not undergoing concurrent chemotherapy showed significantly smaller changes in PLGF values post-treatment (median 0.01 ng/mL, IQR 0.006–0.03) compared to patients treated with chemotherapy (median 0.11 ng/mL, IQR 0.078–0.18, *p* = 0.02). The association with changes in PLGF values was also no longer significant after adjusting for multiple testing. Similarly, two out of three patients treated with radiation alone demonstrated relatively stable levels of Ang1 and VEGF following treatment, although with the limited number of patients treated with radiation alone the effects of chemotherapy on changes in Ang1, Ang2, or VEGF were not significant as compared to the overall population studied (*p* = 0.36, 0.13, and 0.63, respectively, Additional file 1: Table S2, Additional file 1: Figure S1). There were there no baseline differences in cytokine levels between the chemotherapy and no chemotherapy groups. Additionally, the significant changes we observed over the course of therapy for all cytokines measured remained significant excluding the patients who did not receive chemotherapy from the analysis (*p* < 0.0001 for all cytokines).

We also examined potential correlations between serum cytokines and circulating CD4+, CD8+ and CD14+/HLA-DR- MDSC cells (Additional file 1: Table S3). There were no significant correlations between the observed trends in cytokines and these immune subpopulations.

To further confirm our findings and investigate the time course of cytokine changes that we observed, an additional 16 consecutive HNSCC patients undergoing chemoradiation were tested over 4 time points during therapy: the beginning (week 1), middle (week 4), end (week 6–7) of therapy, and in follow-up (approximately 7 weeks following the completion of chemoradiation). Evaluation of these additional patients revealed that VEGF and Ang1 levels decreased from the beginning to the end of treatment in 11/16 (69 %) and 15/16 (94 %), respectively. In these patients that demonstrated a decrease, the middle time point demonstrated a consistent decreasing trend in 9/11 (82 %) and 9/15 (60 %) of cases, respectively, suggesting a constant decrease in these angiogenic cytokines over the course of therapy. The follow up time point demonstrated a more variable trend, with further decreases in VEGF and Ang1 observed in 6/11 (55 %), and 5/15 (33 %).

In regards to Ang2, we observed increases in 13/16 (81 %) of the additional patients, and in 8/13 (62 %) of these the middle treatment time point supported a consistent increase. In 8/13 (62 %) of these patients, the increase in Ang2 continued after treatment was completed in to the follow up time point.

## Discussion

In this study, we prospectively evaluated a group of HNSCC patients with gross disease undergoing definitive radiation therapy with or without concurrent chemotherapy. These patients had increased levels of circulating angiogenic cytokines as compared to healthy control donors. We measured the change in circulating angiogenic cytokines before and after treatment and found that levels of circulating Ang1 and VEGF significantly decreased over the course of treatment whereas Ang2 and PLGF levels increased. These changes were significant even after adjusting for multiple testing and were relatively consistent across demographic and treatment variables, although the number of patients treated with radiation alone was limited and therefore the applicability of our results to this subgroup of patients is unproven. Interestingly, despite the fact that the number of node negative patients was relatively small in our study, we also found that the increases in Ang2 and PLGF could potentially be greater in the setting of nodal positivity.

Expression levels of angiogenic cytokines have potential prognostic relevance across various malignancies, and can impact response to immunotherapy. A meta-analysis of 47 studies (3476 patients) indicated that VEGF overexpression in the tumor predicts poorer overall survival and progression free survival for patients with head and neck cancer, and also correlates with high risk of lymph node metastases [27], however, the relationship between circulating VEGF levels and outcome are not as straightforward [28, 29]. Moreover, high levels of serum VEGF in patients with advanced melanoma was associated with poorer overall survival when treated with ipilimumab (anti-CTLA4 antibody) and also predicted lack of response to high-dose IL-2 therapy [30, 31].

PLGF and Ang2 are also implicated in pathologic angiogenesis, and increases in these and other angiogenic cytokines may help compensate when VEGF expression is decreased or blocked [32]. Systemic upregulation of circulating levels of PLGF have been observed after antiangiogenic therapy (i.e, bevacizumab) in several malignancies, suggesting that changes in PLGF could promote escape from treatments that inhibit angiogenesis [33]. We extend these findings to patients with head and neck cancer treated with radiation, providing potential mechanistic insights to the changes we observe. Elevated PLGF levels are associated with disease progression and higher risk of lymph node or distant metastases in colorectal, gastric, breast, lung, thyroid cancers, and oral SCC [34, 35]. In vitro models of oral squamous cell and larynx carcinoma have shown that PLGF increases expression of matrix metalloproteinases that promote pathologic neovascularization, and that PLGF inhibition slows this process [36, 37]. Similar to VEGF, PLGF may also inhibit dendritic cell maturation by signaling through VEGFR1 [38]. Serum levels of Ang2 were elevated at baseline in 143 breast cancer patients compared to 100 healthy controls, and the 5-year overall survival was significantly lower in the Ang2 high expression group compared to patients with low serum Ang2 [39]. With respect to HNSCC cancers, Ang2 expression was increased in 85 oral SCC patients compared to 37 controls, and was associated with worse survival at 5-year follow up [40]. Ang2 also has important effects on Tie2 expressing monocytes that may affect antitumor immunity [41], with some long-term responders to GM-CSF based cellular vaccines delivered with or without CTLA-4 blockade developing functional Ang-2 blocking antibodies over time [42]. Antibodies to multiple angiogenic cytokines including Ang2 were also found to be associated with improved survival following hematopoetic stem cell transplantation and GM-CSF based vaccine administered to treat leukemia [43].

Previous studies have indicated that chemoradiation therapy has complex effects on the tumor microenvironment that may significantly impact angiogenesis and anti-tumor immunity. Forty-six patients with esophageal cancer treated with concurrent chemoradiation to 60–64 Gy showed significant decreases in serum VEGF levels, with the changes correlating with overall survival at two-year follow up [44]. Twenty non-Hodgkin’s lymphoma patients treated to median 26 Gy also showed serum VEGF levels decline post-RT, as did 37 patients with pharyngeal and laryngeal SCC treated to a total dose of 40 Gy [24, 45]. Prior animal studies have examined the importance of VEGF in tumor associated angiogenesis and tumor growth, and the expression of this cytokine by head and neck squamous cell carcinoma lines driven by MEK-MAPK and IKK-NF-Kb pathways [46]. Studies on the effects of radiation on Ang1 or Ang2 are much more limited, but a preclinical model treated to 10 Gy whole-brain irradiation led to significant decreases in VEGF, Ang-1 and Tie-2 tissue expression but increased Ang-2 expression [47]. This was intimately associated with the inhibition of endothelial cell proliferation and increased levels of endothelial cell apoptosis, and blocking Ang2 in this setting has led to benefit in preclinical models [48, 49]. The functional role of increased levels of PLGF is controversial, with some suggestion that systemic upregulation may promote resistance to anti-VEGF therapy as mentioned above, and others suggesting that increased PLGF may actually functionally impair angiogenesis and predict response to antiangiogenic therapy [50]. Our results were consistent with the prior studies that demonstrate that radiation is associated with decreases in VEGF and Ang1, and an increase in Ang2; these findings are potentially consistent with endothelial cell apoptosis as well as complex effects on angiogenesis and anti tumor immunity that need to be further explored in preclinical models that can better elucidate the functional consequences of the effects that we have observed.

There are several potential mechanisms that explain the observed trends in circulating angiogenic cytokines. Decreases in VEGF may parallel tumor response or decreases in tumor associated macrophages. Tumor debulking caused by chemoradiation could also contribute to VEGFR1 shedding from tumors, that could then bind VEGF and result in decreased VEGF detected in serum. However, free VEGF may be the more biologically active form. Chemoradiation induced endothelial cell apoptosis can cause increases in Ang2 and decreases in Ang1 as the vasculature remodels. Tissue injury has been shown to directly increase Ang2 mRNA expression in mouse models [51]. Increases in PLGF could also be the result of compensatory secretion from stressed fibroblasts within the radiation field, as has been previously suggested [52, 53], and the relationship between Ang2, PLGF increases and lymph node involvement might be due to patients with nodal disease receiving radiation to larger fields. Angiogenic cytokines also interact with one another, and the combination of parallel trends across multiple cytokines presented here is likely more informative than examining individual cytokines in isolation. For example, PLGF can form heterodimers with VEGF and limit VEGF:VEGF homodimerization. In some cases, increased PLGF has been shown to be a positive marker with Ang2 for vessel normalization inhibiting tumor metastasis. Future mechanistic studies are needed to elucidate the exact mechanism underlying the trends observed.

There are limitations to our study. We prospectively included twenty-four consecutive patients from a single institution and then tested an additional 16 patients across 4 time points. However, analyses in larger cohorts, ideally with more time points evaluated would be helpful to further validate our findings and allow for more subgroup analyses and provide additional insights as to the biology underlying the changes we observed. Although we attempted to limit variability in processing of samples and specifically freeze-thaw cycles, we cannot exclude this as a potential confounder. We selected a relatively homogenous group of patients for this analysis; therefore we are unable to discern dose-dependent effects or impacts across a diverse range of histologies. The majority of patients were treated with chemoradiation; therefore, impacts in patients treated with radiation alone should be explored in more detail. However, the similar underlying pathologies and uniform treatment protocols does allow us greater confidence in interpreting the trends observed between individual patients. Node negative and radiotherapy only subgroups are relatively small, and therefore, associations with these factors should be interpreted with caution. None of our patients were treated with anti-angiogenic therapies (i.e., bevacizumab), so it not possible for us to determine synergies that may exist with combination treatments. Finally, because we enrolled a disease population with very good prognosis our purpose was not to draw conclusions about associations between angiogenic cytokine levels and disease-specific outcomes. Instead, our results may provide insight into systemic effects of focused chemoradiation that may impact response to other therapies target the tumor vasculature or systemic antitumor immunity.

Anti-VEGF therapies (bevacizumab) have been explored in head and neck cancer and in combination with immunotherapy. A phase I trial of 10 locoregionally advanced HNSCC patients combining bevacizumab with concurrent chemoradiation (70 Gy, weekly cisplatin) reported a complete response in all patients, with a mean survival time of 61 months [5]. A phase II trial of 46 patients with recurrent or metastatic HNSCC receiving bevacizumab and cetuximab reported an overall response rate of 16 % and median overall survival of 7.5 months [18]. Their analyses of baseline and post-treatment serum samples from 20 patients indicated a decrease in VEGF and an increase in PLGF post-treatment, which is similar to the effects we have observed.

## Conclusion

Our study suggests that localized head and neck chemoradiation significantly impacts circulating angiogenic cytokines, increasing decreasing VEGF and Ang1 and increasing Ang2 and PLGF. These changes may provide insights as to the biology of HNSCC and compensatory angiogenic pathways potentially activated when circulating VEGF levels are decreased, and also suggest a potential mechanism by which focused chemoradiation may impact systemic immunity and T-cell infiltration at distant sites. Ongoing and planned studies continue to examine the role of VEGF inhibition in HNSCC (NCT00588770, NCT01639911), as well as study combining immunotherapy with anti-angiogenic therapy (NCT02210117, NCT02348008, NCT02141542). The results of our study provide additional rationale to support targeting additional angiogenic cytokines such as Ang2 and PLGF, especially when combined with chemoradiation, and potentially continuing these therapies in the adjuvant setting given the continued rise in Ang2 that we observed in the majority of patients. Finally, our study also provides additional rationale for testing targeted radiation in combination with immunotherapy to promote antitumor immune responses.

## Additional file

Additional file 1:
**Table S1.** Associations between angiogenic cytokine levels at baseline and clinical parameters (Wilcoxon Rank Sum *p*-values for discrete; ANOVA *p*-values for continuous variables). **Table S2.** Angiogenic cytokine levels in patients treated with radiation without concurrent chemotherapy (pg/mL). **Table S3.** Correlation between angiogenic cyokines and immune cell populations (ANOVA significance values). **Figure S1.** Changes in cytokine levels in patients treated with radiation without concurrent chemotherapy. (DOCX 183 kb)

## Abbreviations

Ang1: angiopoietin 1
Ang2: angiopoietin 2
HNSCC: head and neck squamous cell carcinoma
HPV: human papilloma virus
IL: interleukin
IMRT: intensity modulated radiation therapy
MDSC: myeloid derived suppressor cells
PLGF: placental growth factor
T-regs: regulatory T-cells
VEGF: vascular endothelial growth factor
