# Supplementary material for: Effects of definitive chemoradiation on circulating immunologic angiogenic cytokines in head and neck cancer patients
Source: J Immunother Cancer. 2016 Jun 21;4:32. doi: 10.1186/s40425-016-0138-9 (PMC4915184; doi:10.1186/s40425-016-0138-9)
Supplement: Additional file 1: — Table S1. Associations between angiogenic cytokine levels at baseline and clinical parameters (Wilcoxon Rank Sum p-values for discrete; ANOVA p-values for continuous variables). Table S2. Angiogenic cytokine levels in patients treated with radiation without concurrent chemotherapy (pg/mL). Table S3. Correlation between angiogenic cyokines and immune cell populations (ANOVA significance values). Figure S1. Changes in cytokine levels in patients treated with radiation without concurrent chemotherapy. (DOCX 183 kb) [file 40425_2016_138_MOESM1_ESM.docx]

Supplemental Tables and Figures

| Supplemental Table 1. Associations between angiogenic cytokine levels at baseline and clinical parameters (Wilcoxon Rank Sum p-values for discrete; ANOVA p-values for continuous variables) | | | | | | | | | | | | |
| --- | --- | --- | --- | --- | --- | --- | --- | --- | --- | --- | --- | --- |
|  | Sex | Age | Tumor Site | HPV | Smoking status | T-stage | Nodal stage | Overall stage | Baseline RBC | Baseline WBC | Baseline Plt Count | Baseline Monocyte Count |
| Ang1 concentration | 0.3 | 0.78 | 0.2 | 0.99 | 0.88 | 0.17 | 0.64 | 0.66 | 0.98 | 0.4 | 0.71 | 0.34 |
| Ang2 concentration | 1 | 0.08 | 0.37 | 0.11 | 0.84 | 0.92 | 0.34 | 0.77 | 0.16 | 0.94 | 0.26 | 0.13 |
| VEGF concentration | 0.06 | 0.86 | 0.05 | 0.28 | 0.28 | 0.03 | 0.64 | 0.17 | 0.64 | 0.7 | 0.18 | 0.11 |
| PLGF concentration | 0.53 | 0.34 | 0.33 | 0.08 | 0.64 | 0.83 | 0.14 | 0.87 | 0.24 | 0.96 | 0.47 | 0.18 |

| Supplementary Table 2. Angiogenic cytokine levels in patients treated with radiation without concurrent chemotherapy (pg/mL) | | | | |
| --- | --- | --- | --- | --- |
| **VEGF** | Concentration at the beginning of RT | Standard Deviation | Concentration at end of RT | Standard Deviation |
| Patient 1 | 774 | 47 | 210 | 34 |
| Patient 2 | 195 | 25 | 150 | 30 |
| Patient 3 | 1161 | 89 | 1160 | 6 |
| **Ang1** |  |  |  |  |
| Patient 1 | 27440 | 571 | 3194 | 397 |
| Patient 2 | 8192 | 965 | 8957 | 1186 |
| Patient 3 | 14255 | 722 | 14330 | 264 |
| **Ang2** |  |  |  |  |
| Patient 1 | 1304 | 128 | 1796 | 167 |
| Patient 2 | 570 | 30 | 219 | 47 |
| Patient 3 | 180 | 17 | 237 | 4 |
| **PLGF** |  |  |  |  |
| Patient 1 | 299 | 23 | 305 | 45 |
| Patient 2 | 236 | 23 | 249 | 8 |
| Patient 3 | 125 | 6 | 155 | 6 |

| Supplemental Table 3. Correlation between angiogenic cyokines and immune cell populations (ANOVA significance values) | | | |
| --- | --- | --- | --- |
|  | CD4+ | CD8+ | MDSC |
| Ang1 | 0.36 | 0.90 | 0.52 |
| Ang2 | 0.70 | 0.15 | 0.21 |
| VEGF | 0.16 | 0.89 | 0.22 |
| PLGF | 0.78 | **0.04** | 0.79 |
